# *N*-(4-Bromo­phen­yl)pyrazine-2-carbox­amide

**DOI:** 10.1107/S1600536810039036

**Published:** 2010-10-09

**Authors:** Marcelle de Lima Ferreira, Marcus V. N. de Souza, Solange M. S. V. Wardell, James L. Wardell, Edward R. T. Tiekink

**Affiliations:** a Fundaçao Oswaldo Cruz, Instituto de Tecnologia em Fármacos - Farmanguinhos, R. Sizenando Nabuco 100, Manguinhos, 21041-250 Rio de Janeiro, RJ, Brazil; b CHEMSOL, 1 Harcourt Road, Aberdeen AB15 5NY, Scotland; c Centro de Desenvolvimento Tecnológico em Saúde (CDTS), Fundação Oswaldo Cruz (FIOCRUZ), Casa Amarela, Campus de Manguinhos, Avenida Brasil 4365, 21040-900 Rio de Janeiro, RJ, Brazil; d Department of Chemistry, University of Malaya, 50603 Kuala Lumpur, Malaysia

## Abstract

The mol­ecule of the title compound, C_11_H_8_BrN_3_O, is close to planar (r.m.s. deviation of all 16 non-H atoms = 0.103 Å), a conformation stabilized by an intra­molecular N—H⋯N hydrogen bond, which generates an *S*(5) ring. In the crystal structure, supra­molecular chains mediated by C—H⋯O contacts (along *a*) are linked into a double layer *via* N⋯Br halogen bonds [3.207 (5) Å] and C—Br⋯π inter­actions [Br⋯ring centroid(pyrazine) = 3.446 (3) Å]. The layers stack along the *b* axis *via* weak π–π inter­actions [ring centroid(pyrazine)⋯ring centroid(benzene) distance = 3.803 (4) Å].

## Related literature

For the anti­mycobacterial activity of pyrazinamide, see: Chaisson *et al.* (2002 ▶); Gordin *et al.* (2000 ▶); de Souza (2006 ▶). For structural studies of pyrazinamide derivatives; see: Baddeley *et al.* (2009 ▶); Howie *et al.* (2010*a* ▶,*b* ▶,*c* ▶,*d* ▶). For the synthesis, see: Wardell *et al.* (2008 ▶); Vontor *et al.* (1989 ▶). For background to halogen bonding, see: Metrangolo *et al.* (2008 ▶); Pennington *et al.* (2008 ▶). For graph-set nomenclature of hydrogen bonds, see: Bernstein *et al.* (1995 ▶). For details of software used to analyse the shape of the mol­ecule, see: Spek (2009 ▶). 
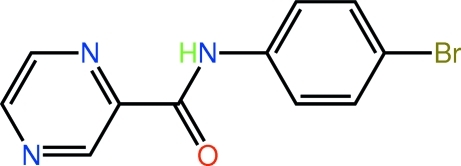


## Experimental

### 

#### Crystal data

C_11_H_8_BrN_3_O
*M* *_r_* = 278.11
Triclinic, 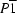

*a* = 5.8396 (4) Å
*b* = 7.3317 (7) Å
*c* = 13.3362 (12) Å
α = 101.670 (4)°
β = 96.728 (5)°
γ = 110.524 (5)°
*V* = 512.55 (8) Å^3^
*Z* = 2
Mo *K*α radiation
μ = 3.99 mm^−1^
*T* = 120 K
0.18 × 0.10 × 0.02 mm

#### Data collection

Enraf–Nonius KappaCCD diffractometer
Absorption correction: multi-scan (*SADABS*; Sheldrick, 2007 ▶) *T*_min_ = 0.764, *T*_max_ = 1.000
8923 measured reflections
2110 independent reflections
1792 reflections with *I* > 2σ(*I*)
*R*_int_ = 0.078

#### Refinement

*R*[*F* ^2^ > 2σ(*F* ^2^)] = 0.054
*wR*(*F* ^2^) = 0.163
*S* = 1.18
2110 reflections
148 parameters
1 restraint
H atoms treated by a mixture of independent and constrained refinement
Δρ_max_ = 0.55 e Å^−3^
Δρ_min_ = −0.52 e Å^−3^

### 

Data collection: *COLLECT* (Nonius, 1998 ▶); cell refinement: *DENZO* (Otwinowski & Minor, 1997 ▶) and *COLLECT*; data reduction: *DENZO* and *COLLECT*; program(s) used to solve structure: *SHELXS97* (Sheldrick, 2008 ▶); program(s) used to refine structure: *SHELXL97* (Sheldrick, 2008 ▶); molecular graphics: *ORTEP-3* (Farrugia, 1997 ▶) and *DIAMOND* (Brandenburg, 2006 ▶); software used to prepare material for publication: *publCIF* (Westrip, 2010 ▶).

## Supplementary Material

Crystal structure: contains datablocks global, I. DOI: 10.1107/S1600536810039036/hb5661sup1.cif

Structure factors: contains datablocks I. DOI: 10.1107/S1600536810039036/hb5661Isup2.hkl

Additional supplementary materials:  crystallographic information; 3D view; checkCIF report

## Figures and Tables

**Table 1:** Hydrogen-bond geometry (Å, °)

*D*—H⋯*A*	*D*—H	H⋯*A*	*D*⋯*A*	*D*—H⋯*A*
N1—H1N⋯N3	0.88 (6)	2.22 (6)	2.708 (7)	115 (5)
C3—H3⋯O1^i^	0.95	2.39	3.177 (8)	140

Symmetry code: (i) 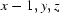.

**Fig. 1.**
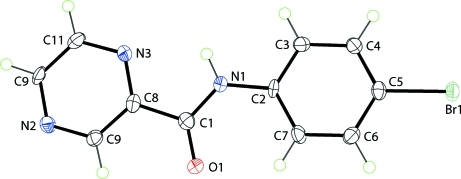
The molecular structure of (I) showing displacement ellipsoids at the 50% probability level.

**Fig. 2.**
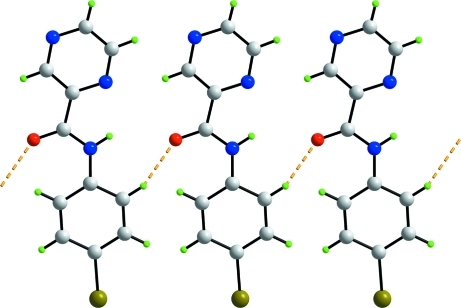
Supramolecular chain in (I) aligned along the a axis. The C—H···O contacts are as orange dashed lines.

**Fig. 3.**
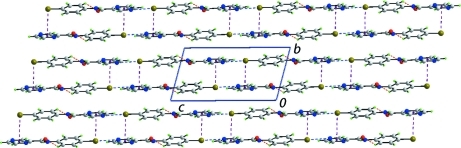
A view in projection down the a axis of the crystal packing in (I) highlighting the stacking of double layers. The C—H···O, N···Br, and Br···π contacts are shown as orange, blue, and purple dashed lines, respectively.

## supplementary crystallographic information

### Comment

Pyrazinamide has well known anti-mycobacterial activity and is the one of the most important drugs used in tuberculosis treatment (Chaisson *et al.*, 2002; Gordin *et al.*, 2000; de Souza, 2006). In continuation of our studies on pyrazinamide derivatives (Wardell *et al.*, 2008; Baddeley *et al.*, 2009; Howie *et al.*, 2010*a*,*b*,*c*,*d*), we report the structure of *N*-(4-bromophenyl)pyrazine-2-carboxamide, the title compound, (I).

The molecular structure of (I), Fig. 1, is essentially planar with the dihedral angle formed between the pyrazine and benzene rings being 10.2 (3)°; the r.m.s. deviation of all 16 non-H atoms = 0.103 Å (Spek, 2009). The observed conformation is stabilized by an intramolecular N—H···N hydrogen bond, Table 1.

An analysis of the crystal packing reveals C—H···O, N···Br, Br···π, and π–π interactions. The C—H···O contacts lead to the formation of a supramolecular chain with a flat topology along the *a* axis. These are sustained in the crystal packing by N···Br halogen bonding [N2···Br1 = 3.207 (5) Å for *i*: 1 + *x*, *y*, 1 + *z*] (Metrangolo *et al.*, 2008; Pennington *et al.*, 2008), as well as Br···π contacts [C5—Br1···*Cg*(N2,N3,C8–C11)^ii^ = 3.446 (3) Å, angle at Br1 = 94.59 (18)° for *ii*: 1 - *x*, 1 - *y*, -*z*]. The resulting double layers stack along the *b* axis, Fig. 3, with the closest interactions between them being of the form π–π [ring centroid(N2,N3,C8–C11)···ring centroid(C2–C7)^iii^ = 3.803 (4) Å, angle of inclination = 10.2 (3)° for *iii*: 1 - *x*, -*y*, -*z*].

### Experimental

*N*-(4-Bromophenyl)pyrazine-2-carboxamide was prepared following the general procedure for *N*-arylpyrazine-2-carboxamides (Wardell *et al.*, 2008). Yield: 48%; m.p. 473 K, lit. value 471–474 K (Vontor *et al.*, 1989). The colourless plate of (I) used for the structure determination was grown from the slow evaporation of its ethanol solution.

### Refinement

The C-bound H atoms were geometrically placed (C—H = 0.95 Å) and refined as riding with *U*_iso_(H) = 1.2*U*_eq_(C). The N-bound H atom was located from a difference map and refined with the distance restraint N—H = 0.88 (1) Å, and with *U*_iso_(H) = 1.2*U*_eq_(N).

### Crystal data

**Table:**

C_11_H_8_BrN_3_O	*Z* = 2
*M**_r_* = 278.11	*F*(000) = 276
Triclinic, *P*1	*D*_x_ = 1.802 Mg m^−^^3^
Hall symbol: -P 1	Mo *K*α radiation, λ = 0.71073 Å
*a* = 5.8396 (4) Å	Cell parameters from 22175 reflections
*b* = 7.3317 (7) Å	θ = 2.9–27.5°
*c* = 13.3362 (12) Å	µ = 3.99 mm^−^^1^
α = 101.670 (4)°	*T* = 120 K
β = 96.728 (5)°	Plate, colourless
γ = 110.524 (5)°	0.18 × 0.10 × 0.02 mm
*V* = 512.55 (8) Å^3^	

### Data collection

**Table:**

Enraf–Nonius KappaCCD diffractometer	2110 independent reflections
Radiation source: Enraf–Nonius FR591 rotating anode	1792 reflections with *I* > 2σ(*I*)
10 cm confocal mirrors	*R*_int_ = 0.078
Detector resolution: 9.091 pixels mm^-1^	θ_max_ = 26.5°, θ_min_ = 3.1°
φ and ω scans	*h* = −7→7
Absorption correction: multi-scan (*SADABS*; Sheldrick, 2007)	*k* = −9→9
*T*_min_ = 0.764, *T*_max_ = 1.000	*l* = −16→16
8923 measured reflections	

### Refinement

**Table:**

Refinement on *F*^2^	Primary atom site location: structure-invariant direct methods
Least-squares matrix: full	Secondary atom site location: difference Fourier map
*R*[*F*^2^ > 2σ(*F*^2^)] = 0.054	Hydrogen site location: inferred from neighbouring sites
*wR*(*F*^2^) = 0.163	H atoms treated by a mixture of independent and constrained refinement
*S* = 1.18	*w* = 1/[σ^2^(*F*_o_^2^) + (0.1*P*)^2^] where *P* = (*F*_o_^2^ + 2*F*_c_^2^)/3
2110 reflections	(Δ/σ)_max_ = 0.001
148 parameters	Δρ_max_ = 0.55 e Å^−^^3^
1 restraint	Δρ_min_ = −0.52 e Å^−^^3^

### Special details

**Table:**

Geometry. All s.u.'s (except the s.u. in the dihedral angle between two l.s. planes) are estimated using the full covariance matrix. The cell s.u.'s are taken into account individually in the estimation of s.u.'s in distances, angles and torsion angles; correlations between s.u.'s in cell parameters are only used when they are defined by crystal symmetry. An approximate (isotropic) treatment of cell s.u.'s is used for estimating s.u.'s involving l.s. planes.
Refinement. Refinement of *F*^2^ against ALL reflections. The weighted *R*-factor *wR* and goodness of fit *S* are based on *F*^2^, conventional *R*-factors *R* are based on *F*, with *F* set to zero for negative *F*^2^. The threshold expression of *F*^2^ > 2σ(*F*^2^) is used only for calculating *R*-factors(gt) *etc*. and is not relevant to the choice of reflections for refinement. *R*-factors based on *F*^2^ are statistically about twice as large as those based on *F*, and *R*- factors based on ALL data will be even larger.

### Fractional atomic coordinates and isotropic or equivalent isotropic displacement parameters (Å^2^)

**Table:**

	*x*	*y*	*z*	*U*_iso_*/*U*_eq_	
Br1	0.20472 (10)	0.24870 (9)	−0.39064 (4)	0.0227 (2)	
O1	0.8942 (7)	0.2880 (7)	0.0884 (3)	0.0259 (10)	
N1	0.4828 (9)	0.2414 (8)	0.0622 (4)	0.0204 (10)	
H1N	0.366 (9)	0.234 (10)	0.099 (5)	0.024*	
N2	0.9106 (9)	0.2715 (8)	0.3965 (4)	0.0223 (11)	
N3	0.4723 (9)	0.2202 (8)	0.2621 (4)	0.0212 (11)	
C1	0.6982 (10)	0.2603 (8)	0.1192 (4)	0.0193 (12)	
C2	0.4304 (10)	0.2444 (8)	−0.0435 (4)	0.0170 (11)	
C3	0.1796 (10)	0.1859 (9)	−0.0902 (5)	0.0211 (12)	
H3	0.0531	0.1461	−0.0512	0.025*	
C4	0.1156 (10)	0.1861 (9)	−0.1937 (5)	0.0215 (12)	
H4	−0.0549	0.1452	−0.2257	0.026*	
C5	0.2979 (11)	0.2453 (8)	−0.2498 (5)	0.0192 (12)	
C6	0.5492 (11)	0.3087 (9)	−0.2035 (5)	0.0210 (12)	
H6	0.6745	0.3519	−0.2427	0.025*	
C7	0.6156 (11)	0.3088 (9)	−0.1011 (5)	0.0205 (12)	
H7	0.7868	0.3525	−0.0694	0.025*	
C8	0.6915 (11)	0.2522 (9)	0.2304 (4)	0.0203 (12)	
C9	0.9064 (11)	0.2777 (9)	0.2980 (5)	0.0198 (12)	
H9	1.0565	0.3004	0.2724	0.024*	
C10	0.6933 (11)	0.2377 (10)	0.4279 (5)	0.0229 (13)	
H10	0.6870	0.2303	0.4979	0.028*	
C11	0.4790 (11)	0.2134 (10)	0.3620 (5)	0.0243 (13)	
H11	0.3300	0.1909	0.3883	0.029*	

### Atomic displacement parameters (Å^2^)

**Table:**

	*U*^11^	*U*^22^	*U*^33^	*U*^12^	*U*^13^	*U*^23^
Br1	0.0268 (4)	0.0286 (4)	0.0140 (3)	0.0123 (3)	0.0025 (2)	0.0066 (2)
O1	0.021 (2)	0.040 (3)	0.019 (2)	0.0123 (19)	0.0067 (17)	0.010 (2)
N1	0.018 (2)	0.031 (3)	0.013 (2)	0.011 (2)	0.0028 (18)	0.005 (2)
N2	0.022 (2)	0.030 (3)	0.020 (3)	0.013 (2)	0.003 (2)	0.012 (2)
N3	0.018 (2)	0.024 (3)	0.017 (3)	0.005 (2)	0.0011 (19)	0.004 (2)
C1	0.020 (3)	0.015 (3)	0.019 (3)	0.005 (2)	0.003 (2)	0.002 (2)
C2	0.025 (3)	0.017 (3)	0.008 (3)	0.006 (2)	0.003 (2)	0.005 (2)
C3	0.016 (3)	0.026 (3)	0.020 (3)	0.008 (2)	0.005 (2)	0.004 (2)
C4	0.019 (3)	0.027 (3)	0.019 (3)	0.011 (2)	0.002 (2)	0.004 (2)
C5	0.026 (3)	0.016 (3)	0.018 (3)	0.011 (2)	0.005 (2)	0.007 (2)
C6	0.023 (3)	0.026 (3)	0.017 (3)	0.011 (2)	0.009 (2)	0.008 (2)
C7	0.018 (3)	0.023 (3)	0.021 (3)	0.007 (2)	0.000 (2)	0.010 (2)
C8	0.026 (3)	0.022 (3)	0.015 (3)	0.011 (2)	0.004 (2)	0.004 (2)
C9	0.022 (3)	0.020 (3)	0.019 (3)	0.010 (2)	0.007 (2)	0.004 (2)
C10	0.029 (3)	0.034 (3)	0.013 (3)	0.014 (3)	0.012 (2)	0.013 (2)
C11	0.024 (3)	0.033 (3)	0.021 (3)	0.012 (3)	0.014 (3)	0.012 (3)

### Geometric parameters (Å, °)

**Table:**

Br1—C5	1.901 (6)	C3—H3	0.9500
O1—C1	1.226 (6)	C4—C5	1.369 (8)
N1—C1	1.337 (7)	C4—H4	0.9500
N1—C2	1.413 (7)	C5—C6	1.394 (8)
N1—H1N	0.88 (6)	C6—C7	1.375 (8)
N2—C9	1.321 (8)	C6—H6	0.9500
N2—C10	1.339 (8)	C7—H7	0.9500
N3—C11	1.340 (8)	C8—C9	1.387 (8)
N3—C8	1.352 (8)	C9—H9	0.9500
C1—C8	1.501 (8)	C10—C11	1.376 (8)
C2—C3	1.398 (8)	C10—H10	0.9500
C2—C7	1.402 (8)	C11—H11	0.9500
C3—C4	1.387 (8)		
			
C1—N1—C2	128.5 (5)	C6—C5—Br1	120.2 (4)
C1—N1—H1N	113 (5)	C7—C6—C5	120.0 (5)
C2—N1—H1N	119 (5)	C7—C6—H6	120.0
C9—N2—C10	116.0 (5)	C5—C6—H6	120.0
C11—N3—C8	115.2 (5)	C6—C7—C2	120.0 (5)
O1—C1—N1	125.4 (6)	C6—C7—H7	120.0
O1—C1—C8	119.5 (5)	C2—C7—H7	120.0
N1—C1—C8	115.1 (5)	N3—C8—C9	121.6 (5)
C3—C2—C7	119.4 (5)	N3—C8—C1	118.2 (5)
C3—C2—N1	117.1 (5)	C9—C8—C1	120.2 (5)
C7—C2—N1	123.5 (5)	N2—C9—C8	122.6 (5)
C4—C3—C2	120.0 (5)	N2—C9—H9	118.7
C4—C3—H3	120.0	C8—C9—H9	118.7
C2—C3—H3	120.0	N2—C10—C11	122.1 (5)
C5—C4—C3	120.1 (5)	N2—C10—H10	119.0
C5—C4—H4	120.0	C11—C10—H10	119.0
C3—C4—H4	120.0	N3—C11—C10	122.6 (5)
C4—C5—C6	120.6 (6)	N3—C11—H11	118.7
C4—C5—Br1	119.1 (4)	C10—C11—H11	118.7
			
C2—N1—C1—O1	1.4 (10)	N1—C2—C7—C6	−179.5 (6)
C2—N1—C1—C8	179.5 (5)	C11—N3—C8—C9	−0.6 (8)
C1—N1—C2—C3	169.0 (5)	C11—N3—C8—C1	179.6 (5)
C1—N1—C2—C7	−13.3 (9)	O1—C1—C8—N3	−179.6 (5)
C7—C2—C3—C4	2.0 (9)	N1—C1—C8—N3	2.2 (8)
N1—C2—C3—C4	179.8 (5)	O1—C1—C8—C9	0.6 (8)
C2—C3—C4—C5	−0.6 (9)	N1—C1—C8—C9	−177.6 (5)
C3—C4—C5—C6	−1.0 (9)	C10—N2—C9—C8	0.4 (9)
C3—C4—C5—Br1	−178.8 (4)	N3—C8—C9—N2	0.3 (9)
C4—C5—C6—C7	1.2 (9)	C1—C8—C9—N2	−179.9 (5)
Br1—C5—C6—C7	179.0 (4)	C9—N2—C10—C11	−0.8 (9)
C5—C6—C7—C2	0.2 (9)	C8—N3—C11—C10	0.2 (9)
C3—C2—C7—C6	−1.8 (9)	N2—C10—C11—N3	0.5 (10)

### Hydrogen-bond geometry (Å, °)

**Table:**

*D*—H···*A*	*D*—H	H···*A*	*D*···*A*	*D*—H···*A*
N1—H1N···N3	0.88 (6)	2.22 (6)	2.708 (7)	115 (5)
C3—H3···O1^i^	0.95	2.39	3.177 (8)	140

Symmetry codes: (i) *x*−1, *y*, *z*.
